# Long-term survival in peritoneal mesothelioma treated with 24 consecutive PIPACs

**DOI:** 10.1515/pp-2024-0034

**Published:** 2025-06-05

**Authors:** Matthias Mehdorn, Boris Jansen-Winkeln, Philipp Rhode, Stefan Niebisch, Yusef Moulla, Till Schönherr, Patrick Sven Plum, Florian Lordick, Rene Thieme, Gertraud Stocker, Maximilian von Laffert, Ines Gockel, Sigmar Stelzner

**Affiliations:** Department of Visceral, Transplant, Thoracic and Vascular Surgery, University Hospital of Leipzig, Leipzig, Germany; Competence Center of Diseases of the Peritoneum, University Hospital Leipzig, Leipzig, Germany; Klinik für Allgemein-, Viszeral-, Thorax- und Gefäßchirurgie, Klinikum St. Georg, Leipzig, Germany; Klinik für Allgemein- und Viszeralchirurgie, Klinikum Chemnitz, Chemnitz, Germany; University Cancer Center Leipzig, Department of Oncology, Gastroenterology, Hepatology, Pneumology, University Hospital of Leipzig, Leipzig, Germany; Institute of Pathology, University of Leipzig Medical Center, Leipzig, Germany; Klinik für Allgemein- und Viszeralchirurgie, Klinikum Magdeburg, Magdeburg, Germany

**Keywords:** malignant peritoneal mesothelioma, PIPAC, long-term survival

## Abstract

**Objectives:**

Malignant peritoneal mesothelioma (MPM) is a rare disease with unspecific abdominal symptoms which is therefore often diagnosed at an advanced stage. Curative therapy is delivered by radical surgery, whereas palliative therapy consists of systemic chemotherapy. Pressurized intraperitoneal aerosol chemotherapy (PIPAC) is a laparoscopically applied chemotherapy which was invented to administer high doses of chemotherapy intraperitoneally in palliative cases of peritoneal malignancies.

**Methods:**

The case of a male patient who received PIPAC treatment as individualized approach for unresectable malignant peritoneal mesothelioma is reported.

**Results:**

The patient began treatment with PIPAC procedures in 2017 for MPM that was unresectable because of extensive disease on the small bowel and refused systemic chemotherapy as the usual standard of care. We initiated PIPAC with doxorubicin and cisplatin and could reach stable disease within one year of treatment so that the therapy was discontinued for 2.5 years. Due to progressive disease, PIPAC was continued resulting in stable disease for 2 years. In total, the patient received 24 PIPAC procedures with no major surgical or toxic side effects over seven years timespan.

**Conclusions:**

We report the case of a patient with MPM who could reach long-term survival of seven years due to a total of 24 PIPAC procedures.

## Background

Malignant peritoneal mesothelioma (MPM) is a rare that arises from the mesothelial layer lining the abdominal cavity. The primary environmental risk factor for the development of mesothelioma is asbestos exposure, which was widely used in industrial products and building materials throughout the 20th century. In addition, several germline mutations, such as mutations in the BRCA gene or BRCA-associated protein (BAP)-1, have recently been linked to the development of malignant mesothelioma [1]. The clinical presentation is unspecific: abdominal pain accompanied by increased abdominal circumference, resulting in delayed and difficult diagnosis of the underlying cause. Large amounts of ascites can be present in more advanced cases [2]. As mesothelial cells may be scarce in ascitic fluid, taking specimen by needle biopsy in case of massive tumor formation or by laparoscopy yields more representative specimen.

Several histological subtypes can be classified: the well-differentiated papillary and the multicystic type with low risk of malignant transformation [1], as well as the malignant epitheloid, sarcomatoid or biphasic type [3]. The epitheloid subtype makes up for about 75 % of all cases [3] and has the best prognosis with a median survival of 55 months [4], while the biphasic type leads to a median survival of 13 months only.

Malignant mesothelioma spreads across the whole abdominal cavity without significant formation of distant metastases or the presence of organ infiltration. Therefore, cytoreductive surgery (CRS) with hyperthermic intraperitoneal chemotherapy (HIPEC) being the curative gold standard. Limitations to complete cytoreduction include extensive involvement of the small intestine, the requirement for major resections, and poor patient performance status. The extent of peritoneal lesions should be quantified and reported using the peritoneal carcinomatosis index (PCI) by Sugarbaker [5] that, by dividing the abdominal cavity in regions and assessing the spread and size of tumor nodules, can yield a maximum of 39 points. Literature reports improved outcomes for peritoneal mesothelioma after successful CRS and HIPEC [1], 6] with a median survival of 61 months [2]. Systemic chemotherapy usually is carried out with cisplatin and pemetrexed, yielding median survival rates of 12 months [1], 3]. In recent years, an increasing role is seen for immunotherapy [7] and further research is being carried out on targeted therapy.

Pressurized intraperitoneal aerosol chemotherapy (PIPAC) is a laparoscopic surgical procedure that generates chemotherapeutic aerosols using nebulizers. The method had been invented in an animal model more than 20 years ago [8] and first its clinical use was reported in 2014 [9] and since has been used in a variety of solid tumors with peritoneal carcinomatosis, especially of gastrointestinal and ovarian origin. By inducing histopathologic regression [10], it can reduce ascites as symptomatic treatment in a palliative setting [11] or might even improve survival [12]. As most of the evidence is based on single center studies, large-scale data by randomized controlled trials for distinct entities is still lacking despite its wide use.

Typically, PIPAC therapy is used either alongside or following the failure of systemic chemotherapy, making it challenging to assess its sole impact on overall survival [13]. We report the case of a patient with MPM who underwent 24 PIPAC procedures without any concomitant chemotherapy and survived for more than seven years following diagnosis.

## Case presentation

**Figure 1:**
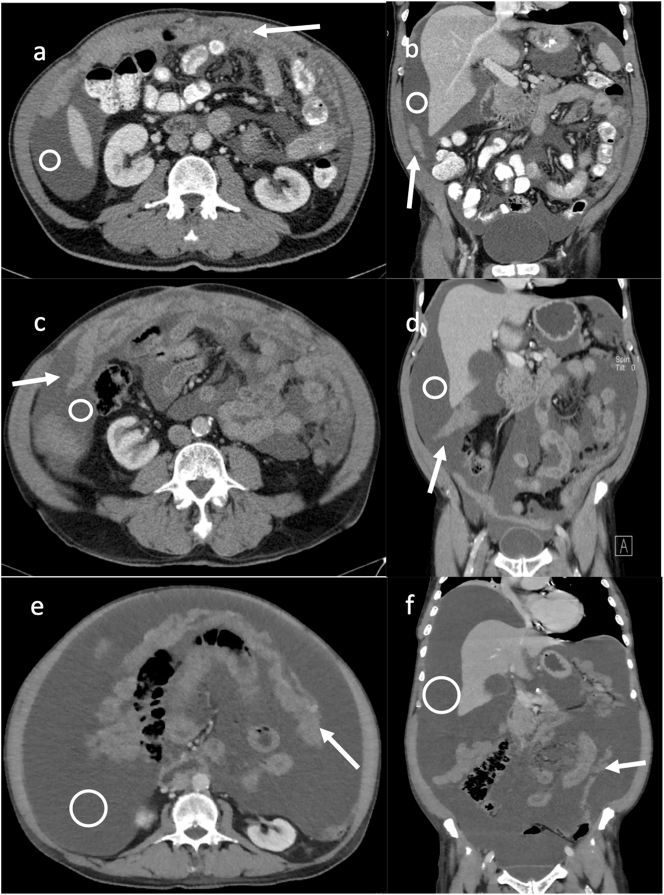
Represented are the contrast enhanced staging CT scans of the patient at three different occasions in axial and coronal slices depicting the ascites (white circle) as well es the omental cake (white arrow) as surrogate for the extent of the disease. (a) and (b) initial CT scan May 2017, (c) and (d) May 2022, (e) and (f) January 2024.

**Figure 2:**
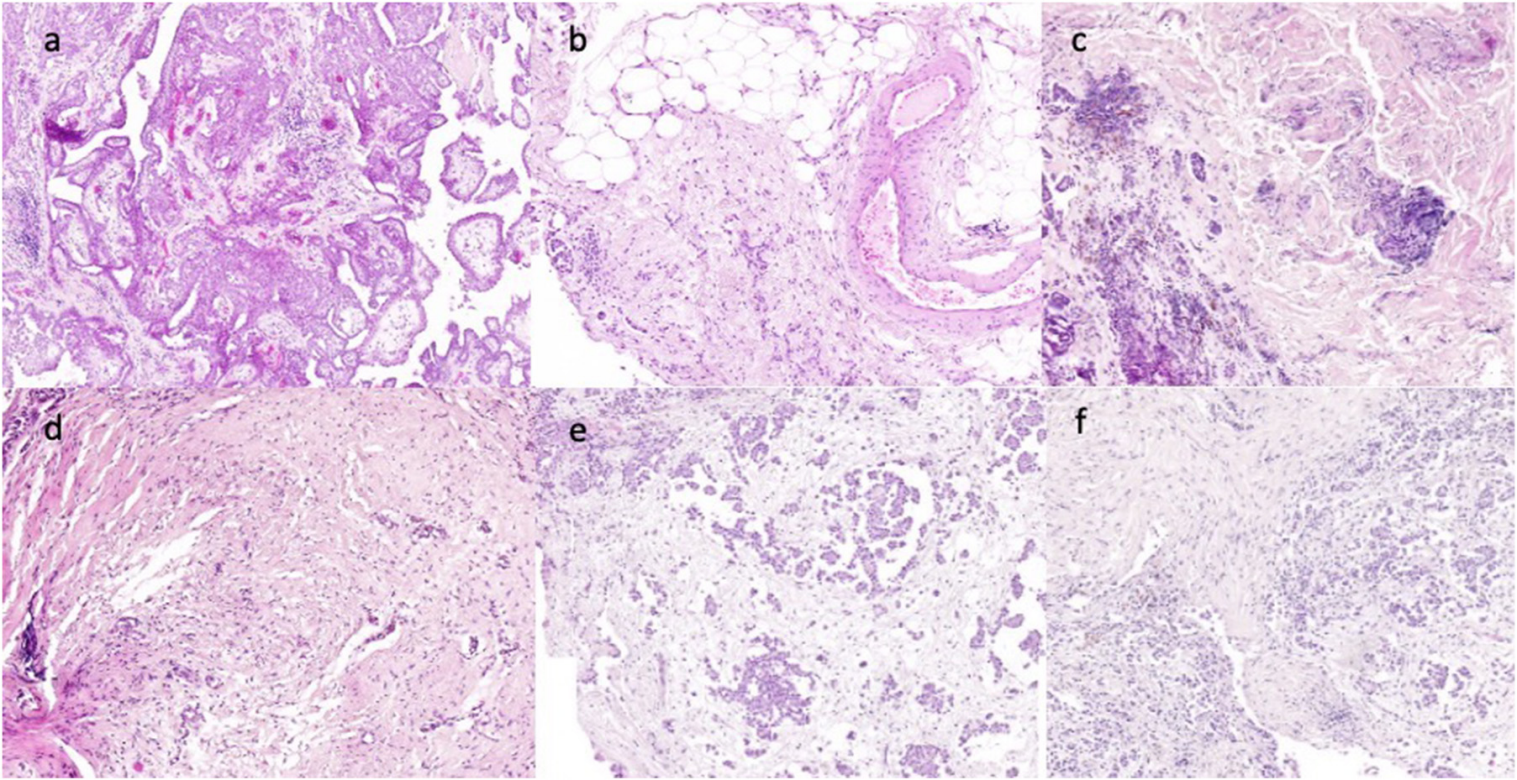
The figure represents histologic specimen from different time points during treatment. (a) Biopsy at diagnosis showing infiltrative epitheloid mesothelioma (5/2017). (b) Biopsy during PIPAC after one year of therapy showing a PRGS 2 (3/2018). (c) Biopsy after restart of PIPAC with pronounced tumor corresponding to PRGS 3 (10/2020). (d) Biopsy after five cycles of PIPAC with dominance of fibrosis and scarce tumor cells PRGS 2 (8/2021) corresponding to reduced ascites. (e) Biopsy during later stages (1/2023) with increasing ascites despite continued PIPAC with PRGS 3 due to more visible tumor infiltration of the fibrotic tissues. (f) Biopsy during penultimate PIPAC therapy with underlying clinical progress (9/2023). Massive tumor collections are present, PRGS4.

We report the case of a 68-year-old male patient who was diagnosed with ascites in 2017. He had no relevant comorbidities and his performance status was Eastern Cooperative Oncology Group (ECOG) 0. CT scans suspected peritoneal carcinomatosis of an unknown primary (Figure 1a and b) and after extensive diagnostic workup a histologic specimen from a diagnostic laparoscopy revealed a papillary, partially epitheloid and infiltrative, thus malignant mesothelioma with a PCI of 18 (Figure 2a). Due to extensive involvement of the small intestine, the patient was deemed ineligible for CRS and the interdisciplinary tumor conference recommended a chemotherapy with cisplatin and pemetrexed in line with institutional standards. However, the patient refused systemic chemotherapy for personal reasons. Thus, as potential alternative treatment in an individualized approach, PIPAC with doxorubicin (1.5 mg/m^2^) and cisplatin (7.5 mg/m^2^) was initiated with palliative intent to control tumor burden and ascites. The patient was enrolled in the institutional PIPAC database with standardized protocols that have been reported before [14], including quality of life questionnaires and standardized peritoneal biopsies from all four abdominal quadrants to monitor histological regression. Therapeutic success was evaluated by PCI, peritoneal biopsies, amount of ascites and repetitive CT scans to exclude systemic progression. Early toxicity was excluded by blood test at day one and three after surgery with further controls by the patient’s general practitioner. Surgical adverse events were queried during readmission for the next PIPAC cycle.

**Figure 3:**
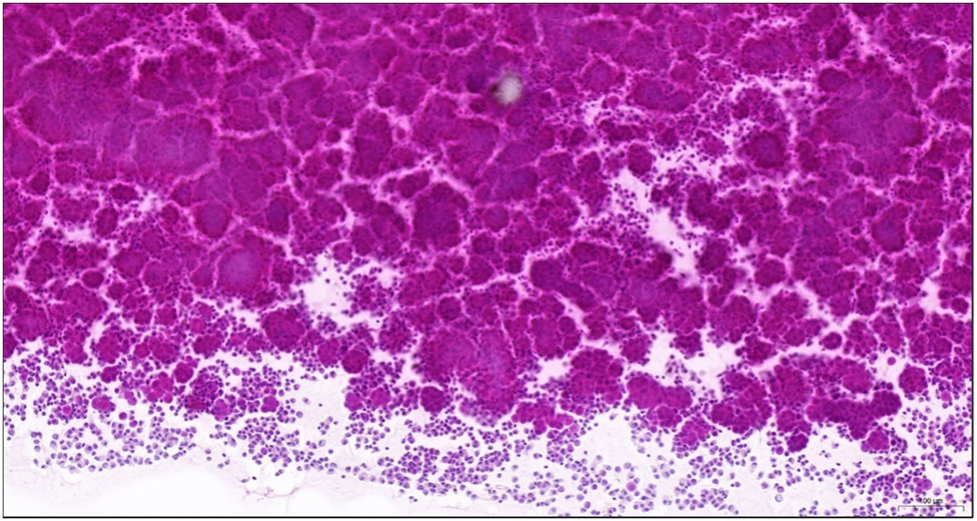
(H & E stained ascites samples): ascites (May 2023) with three-dimensional, polygonal tumorous mesothelial cell clusters, two to three times the size of admixed foamy macrophages (bottom edge of image).

The initial PIPACs were carried out at an interval of six weeks, with larger intervals in the following month due to stable PCI and ascites and histological regression in peritoneal biopsies, classified as peritoneal regression grading score (PRGS) 2 (Figure 2b) [15]. CT scans confirmed the stable disease by exclusion of distant metastases. Together with histologic regression, we recommended follow-up with CT scans every three to six months. The patient remained under the care of his general practitioner for 2.5 years without requiring paracentesis. However, progression of ascites and abdominal discomfort led to reinduction of PIPAC therapy, as the patient still refused systemic chemotherapy. Biopsies at that time showed increased tumor burden (Figure 2c, PRGS 3). Control of ascites was established, starting with a six-week PIPAC interval, stretching it to an interval of 12 weeks which also corresponded with a histologically proven tumor regression (Figure 2d, PRGS 2). For the next two years, the amount of ascites remained stable with the PIPAC therapies (Figure 1c and d). Ascites samples contained mesothelial tumor cell clusters (Figure 3) associated to those were foamy macrophages, whose presence has been linked to chemotherapy induced tumor regression [15].

**Figure 4:**
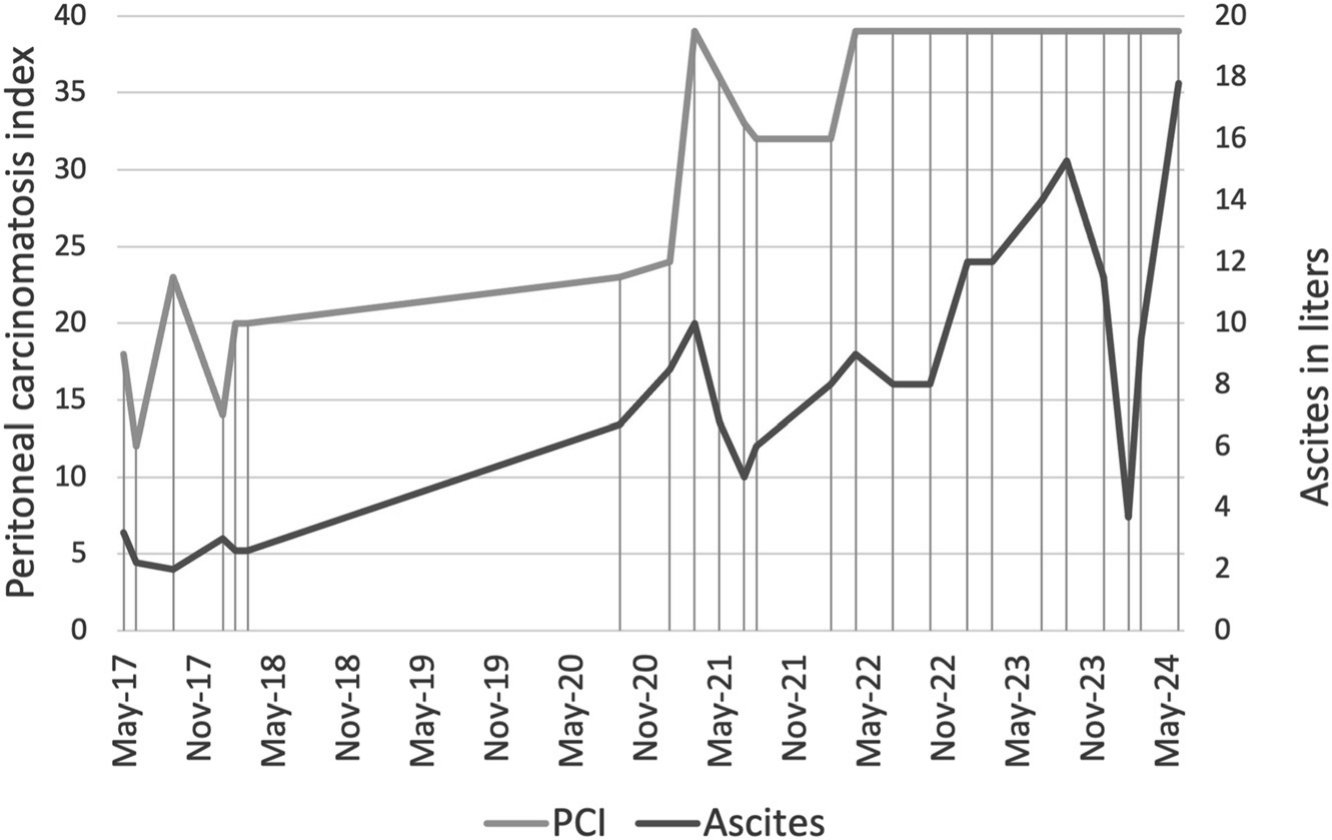
Timeline of the peritoneal carcinomatosis index and the amount of ascites during PIPAC procedures. Vertical lines represent the time points of PIPAC procedures. The dip of the ascites line in recent times was because of paracentesis prior to surgery.

Two and a half years after restart of PIPAC, the ascites volume increased to 15 L, requiring paracentesis (Figure 1e and f). The need for systemic chemotherapy was discussed with the patient several times, but he still refused it, demanding continuation of the PIPAC therapy. In total, 24 PIPAC procedures were performed on this patient. Figure 4 illustrates the progression of ascites and PCI evaluation throughout the course of PIPAC treatments. Ultimately, PIPAC was discontinued due to rapid ascites progression and a decline in the patient’s performance status. The patient’s quality of life, evaluated using the EORTC QLQ-C15 PAL and EORTC QLQ-C30 questionnaires, was completely unaffected by the disease and the treatment. Overall quality of life was rated almost excellent (6 out of 7) and the single items of daily life being not affected throughout most of the treatment period (data not shown). Unfortunately, the questionnaires were not completed for the last four PIPAC procedures, when the patient showed signs of tumor progression such as dyspnea and fatigue.

**Figure 5:**
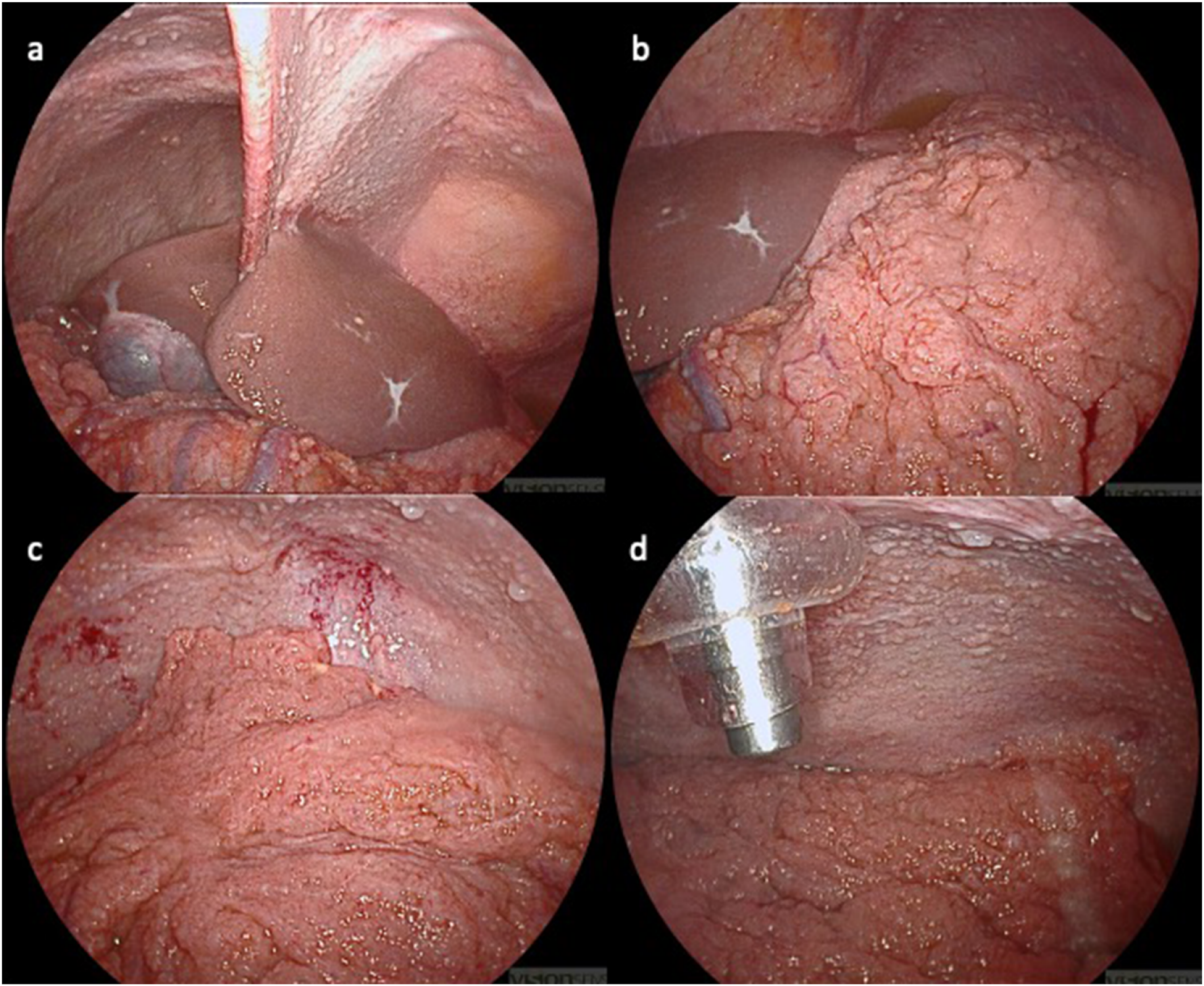
Images of recent PIPAC. (a) Upper right quadrant; (b) upper left quadrant with omental caking; (c) entrance to the pelvis blocked by omental tumor mass; (d) nebulizer introduced via 12 mm trocar. The peritoneal surface is completely covered either with fibrotic peritoneum or mesothelioma nodules.

No major surgical complications, such as bowel injury or chemotoxicity occurred over the course of the repetitive surgeries. The 12 mm trocar site showed a prolonged healing in later stages but did not develop a proper surgical site infection requiring treatment, despite using the same two incisions for each PIPAC procedure. After multiple PIPACs, the peritoneal surface developed a fibrotic aspect, leading to a rigid sensation when introducing the trocar or a needle for paracentesis. This phenomenon has previously been reported after repetitive PIPACs with oxaliplatin [16]. Figure 5 illustrates the abdominal cavity during one of the most recent PIPAC procedures. Transparent nodules represent active tumor. Ascites was amber-colored with a high viscosity, sometimes necessitating intraoperative lavage for complete evacuation.

## Discussion

We present a case of a patient with malignant peritoneal mesothelioma who was treated with a total of 24 PIPACs over a time span of seven years to provide a good control of the disease while maintaining quality of life.

MPM is a malignant disease that is often discovered at an advanced stage due to its unspecific symptoms. Its prognosis is largely depending on the resectability of peritoneal disease and the completeness of cytoreduction (CCR) in cases where cytoreductive surgery and HIPEC are performed. PIPAC is a treatment option for unresectable cases of MPM [17]. According to the annual report of the PIPAC database, only 6 % of PIPACs are performed in patients with MPM, with a reported median survival of 27 months [18]. Another cohort study reported similar median survival [13]. In contrast, our patient survived more than seven years which might have been possible because he was therapy-naïve, preventing the tumor from developing chemoresistance to previous regimens.

Standard chemotherapy in MPM is limited to a few regimens, with cisplatin and pemetrexed being the main combination. This treatment results in a median survival of approximately one year in the pivotal phase-III-study [19]. Other chemotherapy regimen has also been studied but could not provide better survival or safety profiles [2], 3]. Thus, hope is on immunotherapy to improve outcomes for patients with peritoneal mesothelioma, as ipilimumab and nivolumab have provided superior survival rates compared to standard chemotherapy in pleural mesothelioma [20], but also data on PIPAC seems promising [18]. Further evidence for the treatment of MPM using PIPAC may arise in the future by a current study comparing chemotherapy with and without PIPAC [21].

The uniqueness of our case lies in the fact that clinical control of MPM was achieved solely through PIPAC therapy, maintaining low disease activity for several years. Several patient specific factors played a crucial role in the outcome, such as his age, as younger patients have been associated with better prognosis in MPM [22]. Moreover, the histological subtype was partially papillary with an invasive epitheloid component. Despite a rather favorable histologic subtype (epitheloid mesothelioma [6]), one would assume a poor prognosis in unresectable mesothelioma compared to patients undergoing complete cytoreduction [23]. Additionally, the patient repeatedly refused the systemic chemotherapy despite repetitive interdisciplinary consultations. Another key factor was the absence of extra-abdominal metastases as a limiting condition for PIPAC as local intra-abdominal treatment. This combination of factors allowed for a total of 24 repetitive PIPACs with cisplatin and doxorubicin.

Barring various factors suggesting a favorable prognosis, our case presents objective evidence supportring the treatment’s effectiveness. First, histologic regression was induced during the first year of treatment and again upon reinducing the therapy with the same chemotherapeutic agents, as demonstrated by the PRGS (Figure 2). This finding aligns with published data of histologically proven tumor regression in patients with MPM under PIPAC [13]. The increasing PCI in later stages of treatment does not express an ineffective treatment but rather the diffuse peritoneal fibrosis which made macroscopic evaluation of suspicious lesions more difficult [24]. Additionally, ascites remained well controlled alongside tumor regression, so that the patient could be recommended a therapeutic break after one year of induction therapy (Figure 4). Again, the amount of ascites during PIPAC therapy after reinduction was reduced by half after yet another year of therapy. Controlling the ascites burden as symptomatic treatment to ameliorate quality of life has been demonstrated in MPM [13] as well as in other gastrointestinal tumors with peritoneal carcinomatosis [14], 18]. Preserving patients’ quality of life is a key goal of PIPAC therapy. We report QoL data of our patient up until the last year of treatment, showing that he maintained excellent general condition with no significant limitations in quality of life. This is an important finding which stresses the feasibility, efficacy, and acceptance of repeat PIPAC procedures in MPM.

The therapy intensity and the variation of the PIPAC cycles were subject to debate with the patient and within our multidisciplinary tumor board. Several parameters could have been modified: Maintaining the 6-week interval or escalating the dose of chemotherapy in accordance with recent recommendations [25], always bearing in mind possible surgical as well as toxic side-effects.

Although this case reports highlights the potential benefits of PIPAC treatment in peritoneal mesothelioma without systemic chemotherapy, it still is only a case report and thus has its inherent limitations. To evaluate the effect of PIPAC as treatment option in MPM further, large scale well-designed studies are needed and may underline the findings of this case.

## Conclusions

We present a rare case of long-term survival of unresectable malignant peritoneal mesothelioma treated with a total of 24 PIPAC procedures over a period of seven years. For patients who are suitable for an individualized approach involving repetitive laparoscopic surgeries and either are not eligible for or refuse systemic chemotherapy, PIPAC alone poses a viable treatment option in malignant mesothelioma.
